# Gated Resonance Energy Transfer (gRET) Controlled by Programmed Death Protein Ligand 1

**DOI:** 10.3390/nano10081592

**Published:** 2020-08-13

**Authors:** Hubert Grel, Katarzyna Ratajczak, Slawomir Jakiela, Magdalena Stobiecka

**Affiliations:** 1 Department of Physics and Biophysics, Warsaw University of Life Sciences (SGGW), 159 Nowoursynowska Street, 02776 Warsaw, Poland; h.grel7@gmail.com (H.G.); katarzyna_ratajczak@sggw.edu.pl (K.R.); 2 Faculty of Agriculture and Biology, Warsaw University of Life Sciences (SGGW), 159 Nowoursynowska Street, 02776 Warsaw, Poland

**Keywords:** human programmed death protein ligand 1 (PD-L1), resonance energy transfer (RET), gold nanoparticles (AuNP@Cit), gated resonance energy transfer (gRET)

## Abstract

The resonance energy transfer (RET) between an excited fluorescent probe molecule and a plasmonic nanoparticle (AuNP) has been investigated to evaluate the effect of protein molecules on the RET efficiency. We have found that the energy transfer to a functionalized AuNP can be modulated by a sub-monolayer film of programmed death-ligand 1 (PD-L1) protein. The interactions of PD-L1 with AuNP@Cit involve incorporation of the protein in AuNP shell and formation of a submonolayer adsorption film with voids enabling gated surface plasmon resonance energy transfer (SPRET). A model of the gated-RET system based on the protein size, estimated using Fisher–Polikarpov–Craievich density approximation, has been developed and can be utilized for other proteins, with minimum data requirement, as well. The value of the equilibrium constant *K*_L_ determined for the Langmuir isotherm is high: *K*_L_ = 1.27 × 10^8^ M^−1^, enabling highly sensitive control of the gated-RET by PD-L1. Thus, with the gated-RET technique, one can determine PD-L1 within the dynamic range, extending from 1.2 to 50 nM. Moreover, we have found that the Gibbs free energy for PD-L1 binding to AuNP@Cit is −46.26 kJ/mol (−11.05 kcal/mol), indicating a strong adsorption with supramolecular interactions. The proposed gated-RET system, with the fluorescence intensity of the fluorophore probe molecule modulated by plasmonic quenching with AuNP and shielding of energy transfer by the adsorbed PD-L1 can be further developed for determination of PD-L1 in pharmaceutical formulations for immune checkpoint control in cancer therapy.

## 1. Introduction

Significant progress, achieved in the last two decades, in understanding processes that enable cancer to grow and metastasize freely, while evading the immune response of the host, has largely been associated with exploring pathways controlling the apoptosis (e.g., with apoptosis inhibitor proteins, AIP) and the immune checkpoints (e.g., with programmed death protein 1, PD-1). Still, many other proteins may influence the expression of cancer controlling genes and they may also need to be regulated to win the battle with cancer. The use of nanomaterials in cancer research, diagnostics, and therapy is rapidly growing and spans from the targeted drug delivery using nanocarriers [1,2,3,4], to imaging [5,6], including MRI enhancing [7], and to biosensing using various platforms, such as biosensors and sensor arrays [8], microfluidic devices [9,10], and assays [11,12,13], and others. Novel quantum devices and plasmonic effects have recently been discovered, including DNA-linked quantum dots [14,15] and fluorescence enhancement [16,17,18]. In this work, we have focused on the interactions of clinically relevant protein PD-L1, which is a ligand to the receptor PD-1, with a functionalized gold nanoparticle (AuNP). We have found that PD-L1, at low concentrations has the ability to partially block AuNP surface and thus to modulate the resonance energy transfer (RET) from an excited fluorophore probe molecule to the surface plasmons of AuNP. Hence, this property can be utilized for sensitive detection of PD-L1 using gated-RET technique, we have recently developed [19,20].

The programmed death-ligand 1 (PD-L1, also known as CD274 and B7-H1) is a transmembrane protein of 290 amino acids, consisting of immunoglobulin V- and C-like domains, a hydrophobic transmembrane domain, and a cytoplasmic tail of 30 amino acids. The elevated expression of PD-L1 is observed on the surface of tumor cells and associated with worse prognosis for many tumors [21,22,23,24]. PD-L1 is the third member of the B7 family together with B7-1 and B7-2 [25] and it is a ligand for the programmed cell death protein 1 (PD-1), regulating the immune response by suppressing inflammatory activity. It was discovered in 1992, on T cells, by Honjo and his group [26]. Binding of PD-L1 to PD-1 receptors of the immune cells results in the suppression of T cells activity and the evasion of immune reaction of the host [27,28,29,30]. This means that the PD-L1 protein, protruding the surface membrane of a cancer cell, binds to a PD-1 receptor on a T cell and causes the cancer to grow freely. In view of this ability, the PD-1/PD-L1 pathway has been extensively investigated as a predictive biomarker of cancer [27,31,32,33].

The structure of human PD-L1 (hPD-L1) was determined by Garboczi’s group [34] and Chen et al. [35]. The structure of the human PD-1/human PD-L1 complex was reported by Zak et al. [36,37]. The Authors have identified three major hot spots on the surface of hPD-L1 with hPD-1 interaction. Ahmed and Barakat [38] have shown the structural plasticity of PD-L1. They have investigated the conformational space of the IgV domain using principal component analysis (PCA) and molecular dynamics (MD) simulation trajectories.

In our previous paper, we have demonstrated that the resonance energy transfer (RET) from a fluorescein isothiocyanate (FITC) fluorescent dye to citrate-capped gold nanoparticles (AuNP@Cit) can be modulated by a gating sub-monolayer film of cytochrome c (Cyt c) [19], bovine serum albumin (BSA) [19], and survivin (Sur) [20] proteins surrounding AuNP@Cit.

In this work, we have found that PD-L1 can also form protein shells around the AuNP@Cit nanoparticles. Owing to the modulation of the RET between a fluorophore probe molecule (FITC) and a AuNP core, it became possible to determine the value of the equilibrium constant *K*_L_ for the Langmuir adsorption isotherm and the Gibbs free energy Δ*G*°_ads_ for PD-L1 binding to a citrate-capped gold nanoparticle surface. Furthermore, we have been able to utilize the principle of gating of a plasmon-enhanced RET for the detection of PD-L1 at low concentrations. A generalized model of the gated-RET for any protein that binds to a functionalized AuNP has been developed based on the average protein density calculated using Fisher–Polikarpov–Craievich equation [39] which enables estimation of the monolayer surface coverage γ_max_ of a protein on AuNPs.

## 2. Materials and Methods 

### 2.1. Chemicals

CD274 (PD-L1) recombinant human protein, tagged with hIgG1-Fc, was purchased from Sino Biological (Thermo Fisher Scientific, Waltham, MA, USA). This recombinant protein was expressed from a DNA sequence encoding the N-terminal segment (Met 1-Thr 239) of the extracellular domain of human CD274 (NP_054862.1) fused to the Fc region of human IgG1 at the C-terminus. Fluorescein isothiocyanate (FITC), tetrachloroauric acid (HAuCl_4_), sodium citrate dihydrate (Na_3_C_6_H_5_O_7_), dimethyl sulfoxide ((CH_3_)_2_SO), sodium borohydride (NaBH_4_), and other agents were purchased from Sigma-Aldrich and used as received. All chemicals used for investigations were of analytical grade purity. Aqueous solutions were prepared with freshly deionized water with 18.2 MΩ cm resistivity (Millipore, Poland). The RET gating measurements were performed using a 50 mM Na_3_Cit solution with pH 7.4. A stock solution of FITC (20 μM) was prepared in dimethyl sulfoxide (DMSO). All concentrations of added reagents cited in this paper are final concentrations obtained after mixing. Curve fitting was performed using the Simplex algorithm. 

### 2.2. Instrumentation

The fluorescence spectra were recorded using Spectrometer model LS55 (Perkin Elmer, Waltham, MA, USA), with 20 kW pulsed Xenon light source and a photomultiplier tube detector. The excitation and emission slit widths were set to 5.0 nm and scan speed to 500 nm/min. The excitation wavelength was set to *λ*_ex_ = 495 nm. The transmission electron microscopy (TEM) images of AuNP@Cit were recorded in the Electron Microscopy Platform of the Mossakowski Medical Research Centre of the Polish Academy of Sciences in Warsaw, Poland. The electronic structure of FITC was calculated using density functional theory (DFT) method with 6-311G* basis set embedded in Wavefunction Spartan 14.

### 2.3. Procedures

The spherical citrate-capped gold nanoparticles (AuNP@Cit) were synthesized using the borohydride-citrate method as reported earlier [40,41,42,43]. Briefly, 2.56 mL of 10 mM tetrachloroauric acid solution was mixed with 9.6 mL of 10 mM sodium citrate solution and poured to 88 mL of deionized water. Next, 8.9 mL of 5 mM sodium borohydride solution was added dropwise, followed by stirring for 30 min, at which time the solution color changed to ruby red. The obtained citrate-capped gold nanoparticles were stored at 4 °C. The concentration of gold nanoparticle solution was determined from exact amounts of reagents used in synthesis. The size of AuNP@Cit was determined from TEM images. The mean diameter of spherical AuNP@Cit was 4.9 ± 0.1 nm (*n* = 142 particles).

## 3. Results and Discussion

### 3.1. Fluorescence Emission Spectra for FITC in the Presence of AuNPs Capped with Sub-Monolayer Films of PD-L1

Among proteins, found in our earlier studies, able to interact with plasmonic AuNPs and modulate quenching of fluorescent probe molecules, were key proteins participating in critical functions in organisms, including protein of the respiratory chain reaction (cytochrome c), anti-apoptotic cancer biomarker (survivin), and a common multi-function systemic protein (albumin). In this work, we have investigated the ability of the immuno-checkpoint protein ligand PD-L1 to perform a modulating functionality in the gated-RET devices. The nanoparticles used in this investigation were citrate-capped gold nanoparticles (AuNP@Cit) and the fluorescent probe was fluorescein isothiocyanate (FITC). Due to the high sensitivity of the gated resonance energy transfer (gated-RET) method, the concentrations of PD-L1 and FITC in the low nM range were applied. 

**Figure 1:**
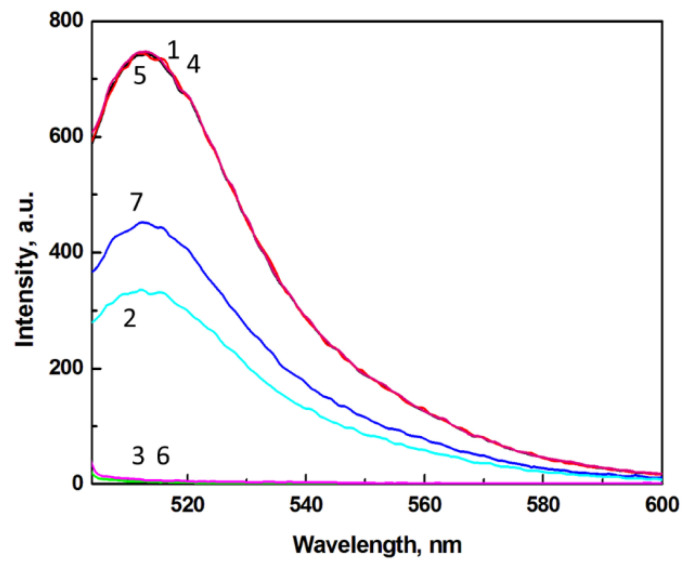
Fluorescence emission spectra for: (1) FITC, (2) FITC + AuNP@Cit, (3) AuNP@Cit, (4) theoretical sum (1) + (3); (5) FITC + PD-L1, (6) PD-L1; (7) FITC + AuNP@Cit/PD-L1; solution: 50 mM Na_3_Cit, pH 7.4, *C*_FITC_ = 66.7 nM, *C*_AuNP@Cit_ = 2.02 nM, *C*_PD-L1_ = 12.7 nM, λ_ex_ = 495 nm.

In Figure 1, fluorescence emission spectra for solutions of FITC probe, AuNP@Cit plasmonic NPs, and PD-L1 modulator, are presented. All spectra were obtained for the excitation wavelength *λ*_ex_ = 495 nm. Curve 1 represents the emission spectrum for FITC alone and shows a well-defined emission peak at *λ*_max_ = 513 nm with intensity of 742.7 a.u. The interactions of FITC molecules with AuNP@Cit nanoparticles result in strong quenching of FITC fluorescence. At the AuNP@Cit concentration level of 2.02 nM and FITC concentration of 66.7 nM, the fluorescence intensity of FITC is quenched down to 328.8 a.u. (curve 2), i.e., by ca. 56%. This confirms that AuNP@Cit nanoparticles are excellent fluorescence quenchers for FITC. The concentrations of AuNP@Cit and FITC dye were chosen based on our earlier studies [19] in which the energy transfer efficiency was optimized. For the sake of comparison of the screening efficiency of different proteins, we keep these conditions unchanged. Thus, the gold nanoparticles at concentration 2.03 nM, causing nearly half of the fluorescence of a 66.7 nM FITC quenched, were applied in this work. Since the solution of AuNP@Cit does not show any emission (curve 3), the fluorescence quenching of FITC must be attributed to the surface plasmon resonance energy transfer (SPRET) from FITC acting as the donor to the surface plasmon on a AuNP@Cit acting as the acceptor. The observed fluorescence quenching of FITC by gold nanoparticles may also have resulted from inner filter effect (IFE) [44,45,46]. As shown in our previous investigations [19], the linear dependence of FITC fluorescence emission intensity on FITC concentration extends from 0 to 70 nM, with slope *ε* = (∂_IFL_/∂*C*_FITC_) = 1.392 × 10^10^ M^−1^ (dependent on the instrumental settings). Upon the addition of gold nanoparticles, the Stern–Volmer plot of *I*_0_/*I* vs. *C*_AuNP_:

(1) $$\frac{I_{0}}{I}=1+K_{SV}C_{AuNP}$$

was employed to determine the Stern–Volmer quenching constant *K*_SV_. In Equation (1), *I*_0_ and *I* stand for the fluorescence intensity in absence of the quencher (AuNP@Cit) and in its presence, respectively. The high value of the *K*_SV_ determined, *K*_SV_ = 1.32 × 10^9^ M^−1^, indicates that the quenching efficiency of AuNP@Cit for FITC is very high [19] and thus, the FITC–AuNP system is very useful for determination of proteins at low concentrations. In the present work, we have tested the effect of PD-L1 on the SPRET from FITC to AuNP@Cit. As seen in Figure 1, curve 5, the addition of PD-L1 at the concentration of 12.7 nM does not quench the emission of FITC at 66.7 nM. Also, a solution of PD-L1 alone does not show any fluorescence (curve 6). However, when PD-L1 is injected to a solution of AuNP@Cit nanoparticles, the fluorescence of FITC is quenched to 449.4 a.u. (curve 7), i.e., by ca. 39%. This quenching is clearly weaker than that observed in the absence of PD-L1. It means that PD-L1 acts as to diminish FITC quenching by adsorbing on the surface of citrate-capped gold nanoparticles, forming a sub-monolayer protein shell (AuNP@Cit/PD-L1), thus modulating the resonance energy transfer between FITC and AuNP@Cit. 

### 3.2. Analysis of the Overlap of FITC Emission Band with Plasmonic Absorption Band of AuNP@Cit 

**Figure 2:**
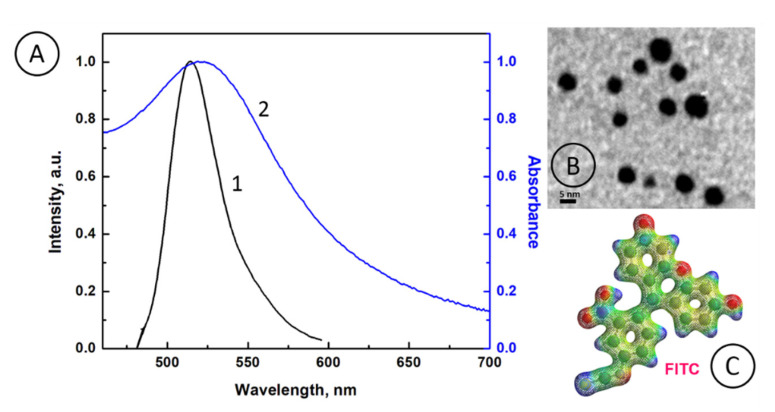
(**A**) Normalized fluorescence emission spectrum of FITC (1) and absorption spectrum of AuNP@Cit (2); (**B**) transmission electron microscopy (TEM) image of citrate-capped gold nanoparticles (AuNP@Cit); (**C**) The electron density surface of FITC structure with color coded electrostatic potential map (from red—more negative to blue—more positive); *ρ* = 0.1 au^−3^.

In Figure 2A, a normalized fluorescence spectrum for FITC donors (curve 1) and a normalized absorbance spectrum for AuNP@Cit acceptors (curve 2), are presented. It is seen that the FITC emission band (curve 1) is fully overlapped with the absorbance band of AuNP@Cit (curve 2), indicating that the probability of a SPRET process between FITC donor and AuNP@Cit acceptor is extremely high. Also, due to large spectra overlap, the fluorescence quenching of FITC by AuNP@Cit may be induced by IFE. In Figure 2B, the transmission electron microscopy (TEM) image of spherical AuNP@Cit particles is presented with their mean particle diameter *d* = 4.9 ± 0.1 nm. The electronic structure of the fluorescence dye FITC is shown in Figure 2C.

### 3.3. Modulation of FITC Fluorescence by PD-L1 Gating 

**Figure 3:**
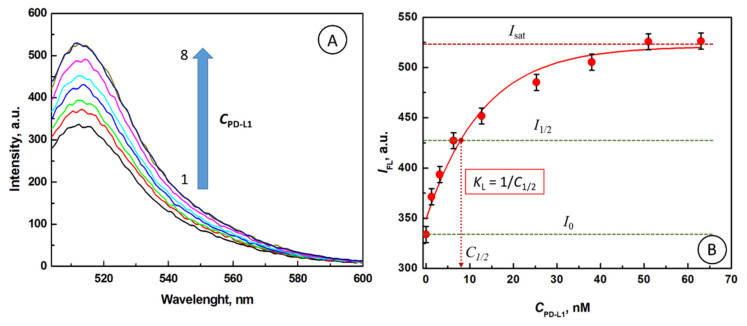
(**A**) Fluorescence emission spectra of AuNP@FITC for different concentrations of PD-L1, *C*_PD-L1_ [nM]: (1) 0, (2) 1.27, (3) 3.17, (4) 6.3, (5) 12.7, (6) 25.3, (7) 38, (8) 51, (9) 63.0. (**B**) The dependence of *I*_FL_ *vs. C*_PD-L1_, *C*_FITC_ = 66.7 nM, *C*_AuNP@Cit_ = 2.02 nM, *C*_PD-L1_ = 0–63 nM, λ_ex_ = 495 nm.

Next, we investigated the gating properties of PD-L1 protein. In Figure 3A, the effect of PD-L1 concentration on fluorescence emission spectra of FITC, following the PD-L1 shell formation on AuNP@Cit surface, is presented. It is seen that the fluorescence intensity of FITC increases from *I*_1_ = 333.8 a.u to *I_2_* = 526.2 a.u., for PD-L1 concentration rise from 0 to 63 nM, respectively. It means that gold nanoparticles quench decreasingly less the fluorescence of FITC with increasing concentration of PD-L1. In Figure 3B, the dependence of *I*_FL_ *vs. C*_PD-L1_ is shown. The results reveal that the PD-L1 is an excellent protein for serving as the gating molecules for the RET process modulation. 

### 3.4. Determination of Model Parameters for PD-L1 Gated Resonance Energy Transfer 

**Figure 4:**
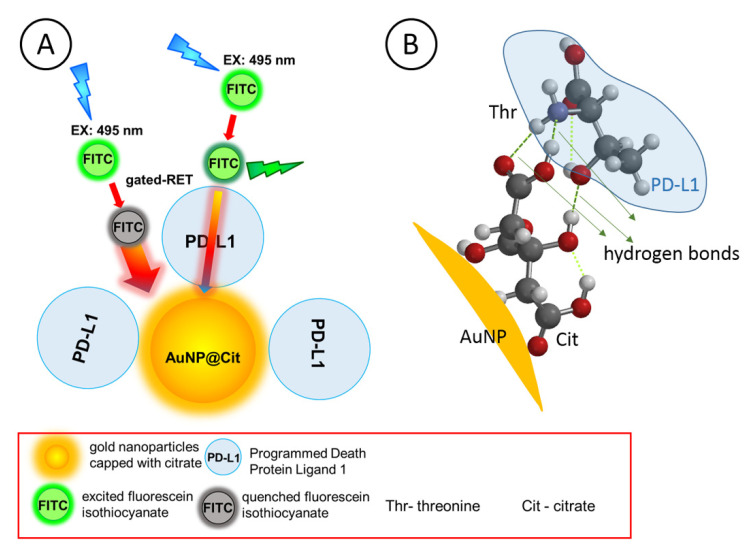
(**A**) Principle of gated resonance energy transfer (gRET) (drawn not to scale); (**B**) Scheme of the interaction of adsorbed citrate (Cit) with threonine (Thr) amino acid of the PD-L1 molecule with the formation of hydrogen bonds.

When a PD-L1 protein at sufficiently high concentration is added to a solution of functionalized AuNPs, a full monolayer adsorption film of that protein is formed. A densely packed adsorption film usually prevents FITC fluorophore probe molecules from directly accessing the AuNP@Cit surface, thus sharply reducing the resonance energy transfer (RET) between the FITC probes and the core AuNP@Cit. However, when a PD-L1 protein at very low concentration is added to a solution of functionalized AuNPs, only a sub-monolayer adsorption film of the protein is formed (AuNP@Cit/PD-L1) and the fluorophore probe molecules are able to enter the voids in the adsorption film (openings, or gates, between protein molecules) and diffuse toward the AuNP surface to interact directly with plasmonic fields of AuNP@Cit. Under such conditions, the SPRET is possible, and its efficiency will be dependent on the surface coverage of protein molecules on AuNP surface. Therefore, by modulating the width of these gates, the RET efficiency can be regulated. In Figure 4A, a generalized model of the gated-RET for FITC and AuNP@Cit/PD-L1 is presented.

In control experiments performed to test the interparticle interactions of AuNP@Cit/PD-L1 using resonance elastic light scattering (RELS) technique, we have found that the RELS intensity initially increases slightly with PD-L1 concentration and then saturates for *C*_PD-L1_ > 2 nM. The maximum increase of RELS, ca. 21.8%, is smaller than that expected for assembly of functionalized AuNPs, therefore it must be attributed to the dielectric function changes occurring in nanoparticle shells due to the protein adsorption/incorporation into the shell and the shell diameter increase.

To estimate the protein surface coverage for this model, for any protein, we can assume a spherical shape for the protein molecules and calculate their maximum surface coverage based on the protein molar mass. The average protein density, needed for these calculations, can be determined using Fisher–Polikarpov–Craievich equation [39]:

*d* = 1.4106 + 0.14528 × e^(−*M*/13.4)^ (2)

where *M* is the protein molar mass [kDa] and *d* is the average density [g/cm^3^]. Thus, for *M* = 52 kDa, one obtains *d* = 1.4136 g/cm^3^. Assuming a spherical shape of PD-L1, one obtains for 5 nm dia. AuNPs, the maximum monolayer surface coverage γ_max_ of PD-L1 on AuNPs as 8.9 pmol/cm^2^. It corresponds to 9.3 molecules of the protein per single AuNP@Cit at close packing density. 

The thickness of the PD-L1 layer was estimated from the volumetric density of the protein and its molecular mass, assuming the spherical shape and a compacted cube shape, as follows.

The molar volume of a protein is given by:

(3) $$V_{mol}=\frac{M}{d}\;\;$$

where *M* is the molar mass of a protein and *d* is the protein density.

Thus, the volume of a single molecule of a protein can be estimated using the equation:

(4) $$V_{molec}=\frac{M}{dN_{0}}$$

where *N*_0_ is the Avogadro number (*N*_0_ = 6.023 × 10^23^ mol^−1^). Assuming spherical shape of the protein molecule, we can estimate the diameter 2*r* of the protein and thus, the monolayer film thickness *h*:

(5) $$\frac{M}{dN_{0}}=\frac{4}{3}\pi r^{3}\;\;$$

(6) $$r=\sqrt[{3}]{\frac{3M}{4\pi dN_{0}}}\;\;$$

(7) $$2r=\sqrt[{3}]{\frac{6M}{\pi dN_{0}}}$$

Similarly, we can estimate the film thickness for protein molecules compacted to cubes with sides *a*:

(8) $$\frac{M}{dN_{0}}=a^{3}\;\;\;\;$$

(9) $$a=\sqrt[{3}]{\frac{M}{dN_{0}}}$$

The ratio of the two values, 2*r* and *a*, is:

(10) $$\frac{2r}{a}=\sqrt[{3}]{\frac{6}{\pi }}=1.24\;\;$$

Hence, if we characterize the film thickness *h* for any protein shape from an ideal sphere to a compacted cube by taking the mean value between 2*r* and *a*:

(11) $$h=\frac{2r+a}{2}$$

the maximum error *E*_max_ in the estimated height *h* of the film will be less than ±10.7%:

(12) $$E_{max}=\left(\frac{2r-h}{h}\right)=\left(\frac{h-a}{h}\right)=\pm 0.107.\;\;$$

For *M* = 52 kDa and *d* = 1.4136 g/cm^3^, one obtains: 2*r* = 4.89 nm, *a* = 3.94 nm, and *h* = 4.41 ± 0.48 nm. A slight modification of this number could be due to the non-regular protein shape, orientation on the surface, and denaturation (if it would occur). 

Another model parameter for gated-RET system is the equilibrium constant for the adsorption of PD-L1 on AuNP@Cit. Assuming Langmuirian adsorption with no self-interactions between the adsorbed PD-L1 molecules, we can express the equilibrium constant *K*_L_ as follows:

(13) $$K_{L}=\frac{\theta }{\left(1-\theta \right)C_{\mathrm{PD-L1}}},$$

where *θ* is the fraction of the AuNP@Cit surface covered by PD-L1 and *C*_PD-L1_ is the concentration of PD-L1 in solution. For such a gated-RET system, the fluorescence intensity of FITC modulated by plasmonic quenching by AuNP and shielding of energy transfer by the adsorbed PD-L1, according to our recent studies [20], is given by:

(14) $$I_{\mathrm{Fl}}=I_{0}+\frac{K_{L}C_{\mathrm{PD-L1}}}{\left(1+K_{L}C_{\mathrm{PD-L1}}\right)}(I_{\mathrm{sat}}-I_{0})$$

This equation describes the modulation of fluorescence intensity of a FITC probe between the intensity *I*_0_ in the absence of PD-L1 and the saturated intensity *I*_sat_ observed for a full monolayer coverage of PD-L1. It is clearly seen from this equation that *K*_L_ is the key parameter controlling the RET behaviour of the system. To determine the value of the Langmuir constant *K*_L_, we note that for:

*K*_L_*C*_PD-L1_ = 1 (15)

Equation (4) reduces to:

*I*_Fl_ = (*I*_sat_ + *I*_0_)/2 = *I*_1/2_ (16)

Therefore, *K*_L_ can be determined graphically from the graph in Figure 3B by reading the value of *C*_1/2_ for the fluorescence intensity equal to *I*_1/2_. The value of *K*_L_ is given by:

*K*_L_ = 1/*C*_1/2_ (17)

Hence, from the graph in Figure 3B, we have: *C*_1/2_ = 7.9 × 10^−9^ M and the value of *K*_L_ is 1.27 × 10^8^ M^−1^. Based on the value of *K*_L_, the Gibbs free energy Δ*G*° for PD-L1 binding to AuNP@Cit can then be calculated using the formula:

Δ*G*° = −2.303 *RT* log(*K*_L_), (18)

where *R* is the gas constant (*R* = 8.3144 J/(mol K)) and *T* is the absolute temperature. For *T* = 298.1 K, the Gibbs free energy of PD-L1 binding to AuNP@Cit is −46.26 kJ/mol (−11.05 kcal/mol). This value of Δ*G*° indicates on a strong adsorption with supramolecular binding.

The surface of PD-L1 protein is predominantly negatively charged at pH = 7.4 of the measurement buffer since the solution pH > p*I*_PD-L1_ = 6.76. The FITC molecules in this solution are deprotonated (p*K*_FITC_ = 6.4) and most of the citrate ions are also deprotonated as well (p*K*_Cit,1_ = 3.14, p*K*_Cit,2_ = 4.77, and p*K*_Cit,3_ = 6.39) [20]. Therefore, the electrostatic repulsions between each other should be observed and binding of PD-L1 to citrate-capped AuNPs should have been prevented. However, on one hand, the electrostatic charges on molecules are considerably screened by the electrolyte with relatively high ionic strength (50 mM citrate buffer), and, on the other hand, supramolecular interactions between PD-L1 and citrate-capped AuNPs are able to overcome the remaining electrostatic repulsion forces. We have previously encountered a similar situation with protein survivin [20], which is also negatively charged at pH = 7.4. However, the adsorption of Sur on AuNP@Cit was observed even at very low Sur concentrations, down to 240 pM, due to the supramolecular interaction forces. In Figure 4B, presented is a simulation of the interactions between a representative amino acid of PD-L1, threonine (Thr), and a citrate-capped core–shell gold nanoparticle (AuNP). It is clearly seen that three hydrogen bonds can be easily formed between peripheral O and N atoms of the citrate and threonine moieties. Therefore, the plausible mechanism of PD-L1 protein binding to gold nanoparticles is likely to be based on the involvement of thiolate bonds, hydrogen bonding, counterions that are screening the excessive negative charges, and cations that are bridging the negatively charged surface functional groups.

Due to the small size of the citrate ligands and their surface mobility in AuNP’s shell, the adsorption of PD-L1 on the surface of AuNP@Cit nanoparticles is random. Since PD-L1 protein contains six cysteine residues in its structure, some of them exposed on its surface, a direct local covalent binding to a gold nanoparticle is also possible after replacement of citrate ions from AuNP surface. However, due to the large PD-L1 size and complex nature of the protein surface, only some of the citrates can be displaced by these cysteine residues. 

### 3.5. Efficiency of PD-L1-Gated Resonance Energy Transfer from FITC to AuNP@Cit/PD-L1_sub-mono_ Ensembles

**Figure 5:**
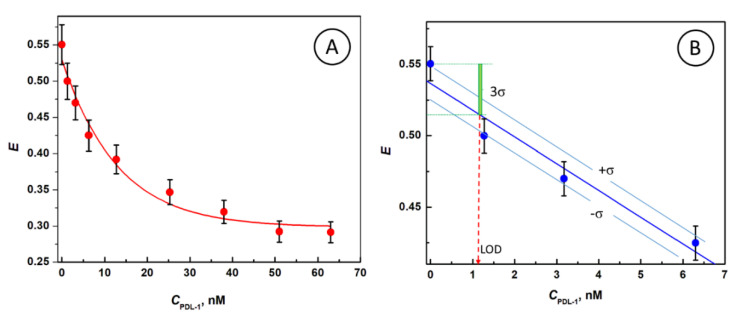
(**A**) Dependence of the gRET efficiency for energy transfer from FITC to AuNP@Cit on PD-L1 concentration showing saturation at higher PD-L1 concentrations. (**B**) Details of the limit of detection (LOD) determination using 3*σ* method (LOD = 1.2 nM). FITC concentration: 66.7 nM, *C*_AuNP@Cit_ = 2.02 nM, *λ*_ex_ = 495 nm.

The quenching efficiency of gated resonance energy transfer (gRET) for different concentrations of PD-L1 protein is shown in Figure 5. The efficiency *E* of gRET is evaluated from the equation:

*E* = (*I*_FL,0_ – *I*_FL_)/*I*_FL,0_ (19)

where: *I*_FL,0_ and *I*_FL_ are the fluorescence intensities for a FITC probe in the absence of AuNP quenchers and in the presence of AuNP@Cit/PD-L1_sub__-mono_ quenchers with different sub-monolayer films of PD-L1, respectively.

The FITC quenching efficiency calculated for AuNP@Cit solution in the absence of PD-L1 is: *E* = 0.55. The protein forming an external shell around AuNP partially blocks the energy transfer process and creates the gates (voids) for controllable FITC access to AuNP surface. At the maximum PD-L1 surface coverage by PD-L1, the gates are minimized, resulting in the residual RET with *E* = 0.31 (Figure 5A). Note that the residual value of *E* greater than 0 is due to the remaining voids between PD-L1 molecules at saturated coverage on AuNP and direct quenching of the probe through the protein if any. This value would be lowered virtually to zero if a protein would undergo a denaturation while binding to a plasmonic host. The non-zero residual RET efficiency at surface saturation with PD-L1 indicates that no denaturation of the protein occurs. The linear part of the graph of *E* vs. C_PD-L1_ for low analyte concentration has been expanded in Figure 5B for the purpose of determination of the limit of detection (LOD) of PD-L1. As seen, the achieved LOD is in the low nanomolar range: LOD = 1.2 nM PD-L1, according to the 3*σ*-method.

## 4. Conclusions

The experiments performed in this work indicate that the immune checkpoint protein ligand PD-L1, in the form of a sub-monolayer adsorption film on a functionalized AuNP, is able to control the gated-RET between a fluorophore probe molecule and a plasmonic core of AuNP@Cit. We have demonstrated a substantial de-quenching of the FITC probe fluorescence by partial coating of AuNP@Cit with PD-L1. The strong adsorption of PD-L1 on citrate-capped AuNPs occurs despite of electrostatic repulsions, as both the PD-L1 and AuNP@Cit are negatively charged at neutral pH. Therefore, the dominant binding forces must be due to thiolate binding and supramolecular interactions. In fact, several hydrogen bonds may be formed between high-oxygen content citrate shell on AuNPs and numerous functional groups on the protein surface. Also, the counterions in electrolyte ensure effective screening of excessive negative charges and cations provide bridging options to negatively charged surface functional groups. The strong supramolecular binding of PD-L1 to AuNP@Cit is corroborated by the high value of the equilibrium constant characterizing the Langmuir adsorption isotherm for PD-L1 on AuNP@Cit: *K*_L_ = 1.27 × 10^8^ M^−1^. A model mechanism of the gated-RET controlled by PD-L1 has been developed. This work demonstrates the interactions of immune checkpoint protein PD-L1 with AuNP@Cit and the high sensitivity of gRET transduction, thus developing the base model for future applications of gRET biosensors with biorecognition capabilities for the detection of proteins in complex real samples in studies of immune checkpoint cancer therapy.
